# Prognostic and predictive biomarker developments in multiple myeloma

**DOI:** 10.1186/s13045-021-01162-7

**Published:** 2021-09-23

**Authors:** Craig T. Wallington-Beddoe, Rachel L. Mynott

**Affiliations:** 1 College of Medicine and Public Health, Level 4, Flinders Centre for Innovation in Cancer, Flinders University, Bedford Park, SA 5042 Australia; 2 Flinders Medical Centre, Bedford Park, SA 5042 Australia; 3 Centre for Cancer Biology, SA Pathology and The University of South Australia, Adelaide, SA 5000 Australia; 4 Adelaide Medical School, Faculty of Health and Medical Sciences, The University of Adelaide, Adelaide, SA 5000 Australia

**Keywords:** Multiple myeloma, Diagnosis, Prognosis, Therapy, Biomarker

## Abstract

New approaches to stratify multiple myeloma patients based on prognosis and therapeutic decision-making, or prediction, are needed since patients are currently managed in a similar manner regardless of individual risk factors or disease characteristics. However, despite new and improved biomarkers for determining the prognosis of patients, there is currently insufficient information to utilise biomarkers to intensify, reduce or altogether change treatment, nor to target patient-specific biology in a so-called predictive manner. The ever-increasing number and complexity of drug classes to treat multiple myeloma have improved response rates and so clinically useful biomarkers will need to be relevant in the era of such novel therapies. Therefore, the field of multiple myeloma biomarker development is rapidly progressing, spurred on by new technologies and therapeutic approaches, and underpinned by a deeper understanding of tumour biology with individualised patient management the goal. In this review, we describe the main biomarker categories in multiple myeloma and relate these to diagnostic, prognostic and predictive applications.

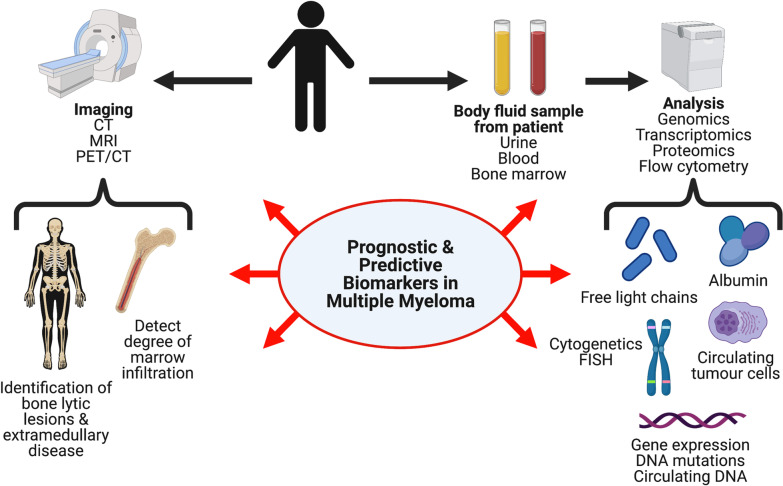

## Introduction

Multiple myeloma (MM) is an incurable haemopoietic malignancy caused by uncontrolled proliferation of neoplastic plasma cells. It is the second most common blood cancer with approximately 140,000 newly diagnosed patients each year worldwide and 1876 new cases diagnosed in Australia in 2018 [1]. Despite recent advances in therapy, the 10-year survival rate remains at only 17% [2]. In MM, a complex array of genetic and epigenetic changes leads to neoplastic transformation of plasma cells, resulting in their uncontrolled growth within the bone marrow (BM) and secretion of large amounts of non-functional monoclonal antibody (known as paraprotein or M protein) into the circulation [3, 4]. The main clinical manifestations of MM are the development of osteolytic bone lesions resulting in bone pain and fracture, hypercalcaemia, renal insufficiency and those relating to bone marrow failure. MM encompasses a spectrum of clinical variants ranging from benign monoclonal gammopathy of uncertain significance (MGUS) and smouldering/indolent MM (SMM) to more aggressive, disseminated forms of MM and plasma cell leukaemia. Whilst the emergence of proteasome inhibitors such as bortezomib and carfilzomib, and immunomodulatory drugs (IMiDs) such as lenalidomide, has seen overall survival (OS) increase from 3 to 6 years in the past two decades, relapse usually occurs. New approaches to better risk stratify MM patients for informing on prognosis and therapy selection are needed since most patients are managed in a similar manner regardless of individual risk factors [1, 4].

According to the NIH Biomarkers Definitions Working Group, a biomarker is defined as “a characteristic that is objectively measured and evaluated as an indicator of normal biological processes, pathogenic processes, or pharmacological response to a therapeutic agent [5].” Biomarkers are found in bodily fluids or other tissues and can be categorised as genomic, transcriptomic, proteomic and clinicopathologic including imaging that can be used for diagnostic, prognostic or predictive purposes [6]. Diagnostic and prognostic biomarkers are essentially what their name states, whilst predictive biomarkers predict clinical outcomes by assisting with therapy selection and optimisation, thereby indicating the likelihood of benefiting from specific therapies [6]. However, despite new and improved biomarkers for determining the overall prognosis of MM patients, there is currently insufficient information to routinely utilise predictive biomarkers to select initial treatment for MM, intensify treatment for high-risk MM, reduce treatment for low-risk MM or for changing to an alternative treatment strategy altogether. In this review, we describe the main biomarker categories in MM, relating these to diagnostic, prognostic and predictive applications.

## Current prognostic staging systems

In 2014, the International Myeloma Working Group (IMWG) revised the definition and diagnostic criteria for MM [7]. The revised definition includes patients who are symptomatic due to the presence of one or more CRAB criteria (hypercalcemia, renal insufficiency, anaemia and bone lesions) together with > 10% clonal plasma cells in the bone marrow biopsy and those who lack CRAB criteria though have either > 60% clonal plasma cells in the bone marrow, a serum involved/uninvolved free light chain ratio of > 100 or more than one focal lesion > 5 mm on MRI imaging of the skeleton [7].

The first staging system by Durie-Salmon included the CRAB criteria and paraprotein level for prognostication; however, there are difficulties in its application due to inconsistencies in the interpretation of the number of bone lesions, whilst elevated serum creatinine and lower haemoglobin levels have aetiologies that may not necessarily relate to MM [8]. In 2005, the International Staging System (ISS) was developed utilising only the prognostic biomarkers albumin and β_2_-microglobulin [9]. The ISS performed well at the time of its creation but could not consistently predict clinical outcomes in the era of novel agents with no differences in OS between staging groups [9]. Moreover, higher values of β_2_-microglobulin and lactate dehydrogenase (LDH) are correlated with inferior survival and associated with greater tumour burden; however, they are also affected by renal failure and other comorbidities which can confound the results and limit the utility of these biomarkers. To overcome these difficulties, the Revised International Staging System (R-ISS) also utilised β_2_-microglobulin and LDH but with the addition of high-risk cytogenetics [10]. The presence of high-risk cytogenetics by fluorescence in situ hybridization (FISH) automatically classifies the patient as Stage III regardless of LDH or β_2_-microglobulin elevation. Response to treatment is generally followed through paraprotein measurements in serum or urine, or through serum free light chains provided the ratio of the involved to uninvolved free light chains is abnormal. The small subset of non-secretory MM that lacks these measurable serum biomarkers is followed by plasma cell percentage and more recently skeletal imaging using MRI or PET/CT [11]. Whilst designed for informing prognosis, unfortunately, none of these staging systems are useful for making therapeutic decisions and thus lack predictive biomarker capacity.

The asymptomatic stages MGUS and SMM are generally characterised by a lower percentage of malignant plasma cells and lower levels of serum M protein, light chains and other traditional diagnostic biomarkers. MGUS is present in approximately 3.5% of the general population over the age of 50 and progresses to MM at the rate of approximately 1% of patients per annum [1, 2]. For SMM, the risk is higher at 10% per annum in the first 5 years, 3% per annum for the subsequent 3 years and 1% thereafter [1, 2]. Up until recently, there were no reliable biomarkers to sub-classify SMM patients into those with a high versus low risk of progression to MM. The Mayo Clinic and Spanish models were developed for more robust prognostication of SMM patients (Table 1) and together proposed new criteria for identifying high-risk SMM [12, 13]. Moreover, the Spanish myeloma group was able to show that high-risk SMM patients treated with lenalidomide and dexamethasone had delayed progression and improved OS compared with observation alone, thus demonstrating a predictive biomarker application of the overall model [14].

**Table 1:** Risk stratification of patients with SMM according to Mayo Clinic and Spanish models [12, 13]

	Risk factors	15-year progression (%)
Mayo Clinic model	Group 1: M protein ≥ 30 g/L + ≥ 10% BM plasma cells Group 2: M protein < 30 g/L + ≥ 10% BM plasma cells Group 3: M protein ≥ 30 g/L + < 10% BM plasma cells	Group 1: 87 Group 2: 70 Group 3: 39

	Risk factors	5-year PFS (%)
Spanish model	≥ 95% abnormal plasma cells Immunoparaesis	Zero risk factors: 72 One risk factor: 46 Two risk factors: 4

BM, bone marrow; FLC, free light chain ratio; PFS, progression-free survival

Recently, a new prognostic model for SMM was introduced with median time to progression for low-, intermediate- and high-risk groups being 110, 68 and 29 months, respectively [15]. Quite simply, only bone marrow plasma cell percentage > 20%, M protein > 20 g/L and free light chain ratio > 20 at diagnosis were identified as important variables in multivariable analysis for the stratification of patients with SMM. The simplicity of this model means it is rapidly growing in popularity as a means to managing SMM patients.

## Heavy and light chains as diagnostic and prognostic biomarkers

Currently, serum free light chain (FLC) levels are measured to assist with MM diagnosis, monitor therapeutic responses, define complete response (CR) and to herald relapse in oligosecretory MM [11]. Moreover, an abnormal involved to uninvolved FLC ratio appears to be an accurate predictor of progression for patients with SMM and of survival and therapeutic response in patients with MM [16–18]. An abnormal FLC ratio before autologous stem cell transplantation (ASCT) predicted early progression afterwards and a one-third reduction in FLC levels within 30 or 60 days predicted a favourable prognosis [19, 20]. The prognostic value of FLC was independent of high-risk translocations such as t(4;14) and t(14;16), although they were positively correlated [21]. As MM is characterized by spatial and temporal clonal heterogeneity, determining serum M protein and FLC levels likely reflects the overall MM disease burden in the body [22]. Moreover, measurement of FLCs may even be better than serum M protein in informing on prognosis. Compared with MM relapse associated with an increase in M protein only, relapse defined as an increase in FLC alone predicted a shorter time to second line therapy, increased risk of progression and mortality [23, 24].

## Diagnostic and prognostic utility of imaging modalities

Bone marrow and other traditional modalities for staging MM do not take into account the spatial heterogeneity of disease and thus imaging techniques are required for obtaining more complete information on disease burden. Skeletal survey using X-rays has long been the method used to identify bone lytic lesions; however, this cannot detect extramedullary disease nor cord involvement with MM and is not sensitive in detecting small lytic lesions [25]. The skeletal survey has essentially been replaced by more sensitive imaging modalities including whole body low-dose CT, MRI and ^18^F-fluorodeoxyglucose (FDG) PET/CT which have now been incorporated into diagnostic and response assessment criteria [25–27]. CT can detect early destructive bone lesions but is unable to detect active MM in areas of prior destruction nor extramedullary sites of MM [25, 28]. Conversely, MRI is sufficiently sensitive to detect early marrow infiltration, can differentiate between benign and malignant osteolytic lesions and is useful for detecting the pattern and extent of bone marrow involvement with MM [29]. When used in asymptomatic MM, identification of at least one lesion, a diffuse infiltrative marrow pattern and 20% or more marrow infiltration predicted progression to symptomatic MM [30]. FDG-PET has similar advantages to MRI; however, results are obtained in a more reasonable time frame, whilst CT shows early bone destruction [26]. Thus, PET/CT appears to be the ideal imaging modality for both disease staging and follow-up [31–33]. The metabolic response and number of focal lesions found on PET/CT in patients with newly diagnosed MM after treatment had independent prognostic value [34, 35]. Therefore, these new imaging modalities are capable of accurately differentiating early-stage MM from MGUS and SMM, for which X-rays usually are negative, and are better at predicting disease progression.

## Bone turnover markers as diagnostic and prognostic biomarkers

These markers can be divided into two categories: collagen fragments released from bone matrix during degradation and enzymes released from either osteoblasts or osteoclasts and can be utilized as non-invasive tools for detecting and assessing skeletal morbidity risk and response to anti-resorptive treatment [36]. Biomarkers reflecting osteoclast-mediated collagen degradation such as N-terminal crosslinking telopeptide of type-1 collagen (NTX), C-terminal crosslinking telopeptide of type-1 collagen (CTX), C-terminal crosslinking telopeptide of type-1 collagen generated by metalloproteinase (ICTP) and deoxy-pyridinoline provide information on the remodelling process and reflect whole body bone turnover [37]. Urinary NTX, serum CTX and serum ICTP levels are elevated in MM patients and correlate with advanced osteolytic bone disease [38–40]. Moreover, urinary NTX and serum ICTP correlate with risk for skeletal complications, progression-free survival (PFS) and OS [39, 41, 42]. Finally, procollagen type-1N-propeptide and procollagen type-1 C-propeptide correlate with new bone formation, whilst receptor activator of nuclear kappa B ligand (RANKL) and osteoprotegerin are also important markers of bone turnover which were found to normalize after ASCT [42–44].

## Genomic biomarkers

### Cytogenetics and FISH

In MM, genetic abnormalities detected by interphase FISH have been used extensively for diagnostic and prognostic biomarker purposes [45]. These abnormalities include copy number variations (CNVs) such as hyperdiploidy (trisomies or tetrasomies of odd numbered chromosomes) and focal or chromosome arm gain or loss, together with translocations involving the immunoglobulin heavy chain locus on chromosome 14 [46]. On FISH studies of bone marrow plasma cells, approximately, 40–50% of MM is characterized by the presence of trisomies, whilst the majority of the remaining cases have a translocation involving the immunoglobulin heavy chain locus [47]. Isolated hyperdiploidy, and in particular trisomies of odd-numbered chromosomes, has been reported to portend a more favourable prognosis except those of chromosomes 17, 18 and 21, whilst gain of 1q, seen in 40% of newly diagnosed patients, is considered poor risk [48]. However, a recent large study demonstrated that only trisomy 3 improved PFS, whilst trisomy 3 and 5 improved OS, overcame the poor prognosis of t(4:14) and improved that of del(17p) [48]. Trisomy 21 was associated with worse OS and further reduced OS when associated with t(4:14) and/or del(17p) [48]. For hyperdiploid standard risk patients, excellent responses to lenalidomide-based therapy are expected, demonstrating both prognostic and predictive biomarker applications, a duality that is not uncommon for other genetic defects in MM [49]. Hypodiploid chromosome 13 or monosomy 13/del(13q) is typically associated with a poor prognosis when detected by conventional cytogenetics; however, the close association with other high-risk genetics such as t(4:14) in 80% of cases makes interpretation difficult [50, 51]. In the era of novel agents, monosomy 13/del(13q) is considered intermediate risk as is t(4;14) due to outcomes with treatment approaching that of intermediate risk disease and these MM patients are considered to do best with bortezomib-based regimens [52, 53]. Loss of 1p affecting *FAF1* and *CDKN2C* genes has also been associated with shortened survival [54].

The site of the immunoglobulin heavy chain locus on chromosome 14q32 is the most involved chromosomal translocation locus in MM. A notable favourable prognostic MM feature is translocation t(11;14), which is associated with higher CD20 expression, lymphoplasmacytic or small mature plasma cell morphology, hyposecretory disease and nuclear cyclin D1 expression and dysregulation [55, 56]. This genetic defect acts as a predictive biomarker and can be targeted with the Bcl-2 inhibitor venetoclax although recent clinical trials in relapsed/refractory MM showed increased mortality due to infection with venetoclax resulting in the FDA placing a partial hold on these studies [57]. The t(4;14) (*FGFR/MMSET*) translocation was once thought to be a poor risk feature, but in the era of proteasome inhibitors has a more favourable outcome [58]. High-risk translocations such as t(14;20) and t(14;16), and del(17p) which affects *TP53*, continue to have poor prognosis despite advances in therapeutics [52, 59]. However, patients with these features were found to do better with triplet therapy (bortezomib, lenalidomide and dexamethasone) compared with intermediate or standard risk disease [60].

Current risk stratification is based on individual cytogenetic abnormalities without consideration of more than one being concurrently present, thus potentially rendering the disease course more unpredictable (Table 2). Kumar et al.reviewed 500 patient FISH analyses and found that only 3% of MM patients had no discernible cytogenetic abnormality [61]. One third of patients were found to have a translocation event with the most common being t(11:14) at 18%. A further 12% had an abnormality in the IgH locus. Trisomies predominated with 57% of patients having at least one chromosomal trisomy and 48% having at least two chromosome trisomies [61]. Monosomy 13 was seen in 47% and only 13% had 17p deletion (as either a deletion or monosomy). The most common overlapping cytogenetic abnormalities were translocations with the presence of another IgH abnormality [61]. Monosomy 13/del(13q) was seen in 57% of patients with a concurrent IgH abnormality and rarely without this abnormality or a trisomy. 36% of patients had both a trisomy and IgH abnormality and p53 abnormalities tended to occur with either a translocation or a trisomy and were rarely seen independently. Good risk cytogenetics have generally been considered to be the trisomies, and poor risk cytogenetics included translocation events and p53 mutations. The presence of high-risk FISH without a trisomy conveyed a poor prognosis with median OS of 3 years. However, the same high-risk FISH with at least one trisomy conferred a standard prognosis [61]. This beneficial effect of trisomy was seen irrespective of the type of high-risk cytogenetic defect (translocation or del(17p)).

**Table 2:** Cytogenetic risk classification of multiple myeloma [45]

Cytogenetic abnormality	Genes affected	Percentage in MM	Prognosis
Trisomies	Odd-numbered chromosomes	40–50	Favourable
Monosomy 13	*RB1*	45–50	Intermediate
1q gain	*CKS1B* and others	35–40	Poor
1p del	*FAM46C*, *CDKN2C* and *FAF1*	30	Poor
MYC 8q24	*MYC*	15–20	Poor
t(4;14)	*FGFR-3* and *MMSET*	15	Poor/Intermediate
t(11;14)	*CCND1*	15	Favourable
17p del	*TP53*	10	Poor
t(6;14)	*CCND3*	5	Favourable
t(14;16)	*c-MAF*	5	Poor
t(14;20)	*MAFB*	1	Poor

### Gene expression profiling

Standard karyotyping and FISH on bone marrow samples is time consuming, relatively insensitive and does not describe the heterogeneity of MM [62]. Global gene expression profiling (GEP) integrates the influence of multiple genetic abnormalities on important cellular pathways associated with proliferation, differentiation, apoptosis and other biologic features in a single signature. The University of Arkansas for Medical Science (UAMS) group was the first to define a 70-gene classifier (GEP70) characterizing seven clusters that identified patients with high-risk genomics with shorter PFS and OS [63]. A total of 351 MM patients, 44 MGUS patients and 12 SMM patients were recruited into the study. Those patients with MM but who had a MGUS-like signature demonstrated improved survival. An increased GEP70 was found to be an independent predictor of risk of progression from precursor stages to MM and correlated well with traditional staging, cytogenetic abnormalities and serum β_2_-microglobulin [63]. This model was subsequently reduced to a 17-gene model that had the same prognostic power. The Intergroupe Francophone du Myélome group developed a 15-gene model that complemented the UAMS model in predicting survival in newly diagnosed and relapsed MM patients which correlated well with the prognoses associated with known cytogenetic abnormalities [64]. The genes included in this study mainly pertained to the cell cycle, specifically regulation of chromosomal segregation, highlighting their known importance in MM biology [65]. Another study constructed a proliferation indices model in which proliferation genes differentially expressed between MM cells and non-malignant plasmablastic cells as well as non-proliferating normal plasma and memory B cells were selected and found to be a useful prognostic tool for event-free and OS in patients with MM treated with high-dose chemotherapy and ASCT [66].

Gene expression profiles obtained from patients with newly diagnosed MM from the HOVON65/GMMG-HD4 clinical trial were used to generated a 92-gene signature (EMC-92) capable of distinguishing high- and low-risk MM patients in both newly diagnosed and relapsed disease settings [67]. Interestingly, many of the gene signatures that have been developed do not appear to have many genes in common, for example, those developed by the UAMS (17 genes), IFM trial (15 genes) and MRC Myeloma IX trial (6 genes) [63, 64, 68]. This discrepancy may reflect variations in treatment strategies or patient selection and different aspects of MM biology. Most recently, a 92 gene signature (SKY92) has been validated as predictive of prognosis in MM patients at any stage of their disease and treatment course with even greater prognostic ability when combined with ISS [69].

### Next generation sequencing

Next generation sequencing (NGS) panels have the potential to detect large and small mutational events from CNVs and translocations to single nucleotide polymorphisms (SNPs). NGS was able to identify 15 recurrent somatic mutations in MM with prognostic value from three large studies (Table 3) [70–72]. These and other studies have demonstrated that the mitogen-activated protein kinase (MAPK) pathway, which includes *KRAS*, *NRAS* and *BRAF* genes, is the most commonly mutated signalling pathway in MM. Mutations affecting the nuclear factor κB (NF-κB) pathway, commonly deregulated in B-lymphoid malignancies, are also common in MM [72]. Moreover, mutations in IMiD target pro-survival genes *IRF4* and *EGR1* confer more favourable outcomes, whilst mutations in DNA repair pathway genes *TP53*, *ATM* and *ATR* are considered poor prognostic markers [73–75]. A prognostic model was developed using data from the UK Myeloma XI clinical trial, ISS and mutations affecting *TP53*, *ATM* or *ATR* and *ZFH4* or *CCND1*; CNVs including del(17p) and amp(1q); and translocations involving t(4;14) and *MYC* [72]. The model showed improved sensitivity compared to ISS alone for the early detection of disease progression and prediction of mortality in patients with high-risk MM. More recently, “double hit” MM, defined as bi-allelic *TP53* inactivation or amplification (> 4 copies) of *CKS1B* (1q21) on a background of ISS stage 3, has been identified, constituting ~ 6% of newly diagnosed MM and carries a markedly poor prognosis with median PFS and OS being 15 months and 21 months, respectively [76].

**Table 3:** Common recurrent somatic mutations of prognostic significance [70–72]

Pathway	% with affected pathway	Gene name	Frequency (%)	Prognosis
MAPK	40	*KRAS*	23	Intermediate
		*BRAF*	20	
		*NRAS*	8	
NF-κB	20	*TRAF*	3	Intermediate
		*CYLD*	2	
		*LTB*	3	
DNA repair	10	*TP53*	9	Poor
		*ATM*	3	
		*ATR*	1	
RNA metabolism	15	*FAM463*	9	Intermediate
		*DIS3*	7	
Plasma cell differentiation	10	*IRF4*	3	Favourable
		*EGR1*	5	

MAPK, mitogen-activated protein kinase; NF-κB, nuclear factor κB

## Proteomics

Whilst numerous individual proteins have been shown to carry prognostic and/or predictive significance in MM, unlike genomic analyses, there are comparatively few protein ‘signatures’ that have been developed for this purpose. Proteomic profiling of bone marrow plasma cells from relapsed and/or refractory MM patients revealed a protein signature associated with proteasome inhibitor resistance. In particular, increased expression of proteasome activator complex subunit 1 (PSME1) in patients not achieving a very good partial response (VGPR) correlated with the observed clinical resistance to bortezomib-based therapy [77]. Another study also examined changes in the proteome that predicted response to either bortezomib or lenalidomide containing treatment regimens [78]. Such proteome changes included those relating to endoplasmic reticulum stress and acute phase response signalling, suggesting responsiveness to proteasome inhibitors or IMiDs, respectively [78]. Proteins involved in the transition of MM to plasma cell leukaemia (PCL) are of great interest considering the extremely poor prognosis of this condition. Such a study was conducted in a single patient demonstrating a metabolic shift towards aerobic glycolysis, as well as a downregulation of enzymes involved in glycan synthesis, potentially mediating altered glycosylation of surface receptors. Enzymes involved in epigenetic modifications were not dysregulated [79]. The extracellular matrix (ECM) is required to support the growth and development of MM cells and an interesting proteomic study examined the composition of the ECM in patients with MGUS, newly diagnosed and relapsed and/or refractory MM, compared to healthy control matrix. The tumour ECM is remodelled at the protein level in MGUS and MM to allow development of a permissive microenvironment with two ECM-affiliated proteins, Annexin A2 (ANXA2) and Galectin-1 (LGALS1), more abundant in MM with high expression associated with inferior OS [80].

Before mass proteomic techniques, many individual proteins have been examined for their prognostic or predictive biomarker relevance in MM, one of which is programmed death-ligand 1 (sPD-L1), which when bound to its receptor PD-1 on T-cells promotes immune tolerance. Soluble PD-L1 in bone marrow plasma post-ASCT predicts PFS and OS and correlates with the bone marrow plasma cell percentage [81]. ASCT can reset the immune microenvironment by lowering PD-L1 expression allowing anti-tumour immunity to progress [81]. PD-1 can be targeted with pembrolizumab although trials combining this agent with IMiDs have not demonstrated a favourable benefit-to-risk profile [82, 83]. There is currently much interest in the therapeutic targeting of B cell maturation antigen (BCMA) for plasma cell directed immunotherapies [84]. Serum BCMA (sBCMA) when elevated was associated with shorter PFS and OS and was independent of renal function and other prognostic markers [84]. Moreover, among patients with non-secretory MM, sBCMA correlated with bone marrow plasma cell percentage and with PET/CT findings [85]. The histone methyltransferase MMSET, over-expressed in t(4;14) MM, regulates the expression of many genes including signalling lymphocytic activation molecule F2 (SLAMF7) [86]. SLAMF7 overexpression was demonstrated in t(4;14) positive MM patients samples and knockdown of SLAMF7 in t(4:14)-positive MM cell lines resulted in anti-proliferative and cytotoxic effects [86]. Furthermore, high serum SLAMF7 levels correlated with aggressive MM and inferior PFS [87]. These and other findings contributed to development of the mAb elotuzumab for patients with relapsed and/or refractory MM [88].

Sometimes the target of a drug, though initially an obvious biomarker choice, may not turn out to be useful, for example, IMiDs such as lenalidomide and pomalidomide target cereblon (CRBN), which has been proposed as a predictive biomarker of response. However, there are conflicting reports in the literature concerning the clinical utility of CRBN and its downstream targets as predictive biomarkers of IMiD response, since downregulation of CRBN is not able to explain a lack of response [89, 90]. On the other hand, immunoglobulin lambda (Igλ) translocations confer a poor outcome to patients receiving IMiDs through a hitherto unknown mechanism [91]. Controversy also surrounds the expression level of CD38 on MM plasma cells and responses to anti-CD38 monoclonal antibodies such as daratumumab, which are efficacious even when cell surface expression is low [4]. Similarly, mechanisms of resistance to proteasome inhibitors are still not well understood, with mutations in proteasome subunits or expression of multi-drug resistance transporters evident in only a minority of cases and of questionable involvement [4, 92–94]. Recently, the poor prognostic impact of desmoglein-2 on the malignant plasma cells of newly diagnosed MM patients has been demonstrated with efforts underway to elucidate its biological role in MM and to develop targeted therapeutics [95].

## Liquid biopsies: circulating tumour cells and DNA

Due to the variable nature of bone marrow involvement in MM, bone marrow biopsy is unlikely to represent the spatial or temporal mutational landscape of the disease. Circulating tumour cells (CTCs) are released from primary tumour or metastatic sites into the bloodstream with higher numbers suggested to be an adverse prognostic finding for MM patients at diagnosis, after ASCT and during relapse [96–98]. Moreover, genomic characterization of CTCs via whole exome sequencing has demonstrated a high concordance in clonal mutations between CTCs and bone marrow paired samples and some sub-clonal mutations were found only in CTCs [99–101]. Thus, CTCs may represent a more complete picture of MM disease burden throughout the body than bone marrow samples obtained from only one region. This may be particularly true for patients with extra-medullary disease. The next generation flow (NGF) assay for minimal residual disease (MRD) detection (see next section) can also be applied to the detection of CTCs [102, 103]. Additionally, an automated approach based on the differential expression patterns of CD19, CD45 and CD38 has been developed for the isolation and enumeration of CTCs (CELLSEARCH, Menarini Silicon Biosystems). Both techniques have been applied in clinical settings; however, they require further testing and standardisation and are currently unsuitable for MRD evaluation by themselves.

In a multi-centre phase III clinical trial, patients with newly diagnosed MM had blood and bone marrow samples collected at several time points including at diagnosis and achievement of CR or suspected CR, and after induction therapy, ASCT and consolidation therapy [104]. Tumour cell quantitation was performed by PCR designed to detect 1 tumour cell in 330,000 mononuclear cells with the lowest detectable number of CTCs being 1 cell in 7.75 × 10^7^ mononuclear cells. A 99.6% reduction in the number of CTCs was seen most significantly from diagnosis to post-ASCT. Interestingly, ISS III patients only achieved a reduction in CTCs after ASCT and not with induction therapy alone. Moreover, there was a direct correlation between the number of tumour cells in the bone marrow if CTCs were detected.

Detection of circulating tumour DNA (ctDNA) in the peripheral blood of patients may avoid the need for invasive bone marrow biopsies in diagnosis and monitoring for progressive disease. CtDNA comprises degraded DNA fragments released into the bloodstream from cancer cells and represents a molecularly distinct DNA fragment of the total cell-free DNA (cfDNA). Recently, using ultra-deep sequencing targeting all protein coding exons of a 5-gene panel for paired ctDNA and bone marrow samples, a 96% concordance rate for detecting tumour-derived mutations with allele fraction as low as 0.25% was obtained [105]. Furthermore, the authors reported three cases with detectable clonal populations in the ctDNA but not in matched bone marrow samples suggesting determination of sub-clones through the analysis of blood plasma may be more informative than that obtained from single bone marrow aspirates. Despite these encouraging findings, the utility of ctDNA for monitoring MRD is controversial with conflicting reports in the literature. For example, one study demonstrated a high correlation between next generation sequencing (NGS) of IgH gene rearrangements in the ctDNA of diagnostic and post-treatment samples with 8-colour flow cytometry [106]. On the other hand, detectable ctDNA of diagnostic samples could only be tracked in 39% of patients with VGPR or a worse response, whilst another study found ctDNA was undetectable in 69% of samples which were clearly MRD positive in the bone marrow [107, 108]. However, it should be noted that ctDNA may decline more rapidly than in other plasma cell compartments [109, 110]. Overall, the use of ctDNA as a tool for MRD monitoring and prognostication is not mature and requires further integration of molecular techniques, bioinformatic analysis and evaluation within clinical trials.

## Measurable (minimal) residual disease

As therapy for MM improves and more patients achieve a stringent complete response (sCR), new highly sensitive techniques are required to detect residual disease. Two techniques have demonstrated the ability to accurately measure residual clonal plasma cells in the marrow, including next generation flow cytometry (NGF, e.g. EuroFlow) and NGS of immunoglobulin genes (e.g. Clonoseq: Adaptive Technologies) [111, 112]. Continually strengthening data suggest that MRD could be used as a predictive and/or prognostic biomarker to evaluate treatment efficacy, inform on therapeutic decision making and predict PFS and OS [113]. Methods to detect MRD include multiparameter flow cytometry (MFC), allele-specific oligonucleotide quantitative PCR (ASO-qPCR) and NGS. The sensitivity of 8-colour MFC is one clonal plasma cell in 10^5^ normal cells although highly standardized assays such as EuroFlow can reach a sensitivity of 1 in 10^6^ [114]. The sensitivity of MFC is highly dependent on bone marrow sample quality, number of cells analyzed and the composition of the antibody panels. ASO-qPCR of variable diverse joining (VDJ) heavy chain rearrangements requires the production of patient-specific probes to detect and amplify clonal DNA regions. Both techniques rely on bone marrow sampling which can markedly affect the quality of MRD detection and the generation of false negative results. NGS allows the detection of clonal VDJ rearrangements with a high sensitivity of 1 clonal cell in 10^6^ normal cells. Originally introduced for patients with acute lymphoblastic leukaemia and chronic lymphocytic leukaemia, NGS has proved to also be applicable to MM and is more sensitive than traditional MFC and ASO-qPCR. Imaging tools such as PET/CT also play an important part in MRD detection, specifically for the detection of extramedullary disease and early tumour activity. The IMWG has defined response criteria categories of MRD negativity to allow uniform and structured reporting (Table 4).

**Table 4:** International myeloma working group MRD criteria [11]

Result	Criteria definition^a^
Sustained MRD negative	MRD negativity in the marrow (NGF or NGS, or both) and by imaging as defined below, confirmed minimum of 1 year apart. Subsequent evaluations can be used to additionally specify the duration of negativity (e.g. MRD negative at 5 years)
Flow MRD negative	Absence of phenotypically aberrant clonal plasma cells by NGF on bone marrow aspirates using the EuroFlow standard operation procedure for MRD detection in multiple myeloma (or validated equivalent method) with a minimum sensitivity of 1 in 10^5^ or greater nucleated cells
Sequencing MRD negative	Absence of clonal plasma cells by NGS on bone marrow aspirate in which presence of a clone is defined as fewer than two identical sequencing reads obtained after DNA sequencing of bone marrow aspirates with the LymphoSIGHT platform (or validated equivalent method), with a minimum sensitivity of 1 in 10^5^ or greater nucleated cells
Imaging plus MRD negative	MRD negativity as defined by NGF or NGS plus disappearance of every area of increased tracer uptake found at baseline or a preceding PET/CT or decrease to less than mediastinal blood pool SUV or decrease to less than that of surrounding normal tissue

MRD, minimal residual disease; NGF, next-generation flow; NGS, next-generation sequencing; SUV, standardized uptake value

^a^These criteria require achieving complete response on the basis of the standard International Myeloma Working Group response criteria [11]

## The clinical utility of MRD

MRD testing in MM is increasingly being used in clinical trials for the assessment of disease response and as a prognostic tool for predicting response duration; MRD negative responses result in improved PFS, whilst remaining MRD positive after treatment confers a higher risk of relapse [115, 116]. Thus, MRD is becoming a relevant surrogate marker for PFS and possibly OS in MM. A small study of 39 patients reported by the Italian MM group was among the first to establish that MRD dynamics could be another relevant prognostic factor [117]. Other clinical studies suggested that MRD kinetics are more informative than single time point assessments and may be useful in addressing specific clinical questions [118].

A very relevant, but somewhat unexplored area of investigation, is the understanding of how monitoring the depth of response in individual patients might inform prognosis and potentially be used to predict response to and guide therapy. A recent study showed that patients achieving a depth of response at a level of 10^−3^ had a projected PFS of 35 to 45 months, whereas patients with a depth of response to the level of 10^−5^ had a projected PFS of > 80 months [119]. This brings into question whether patients who achieve a lesser response (i.e. at a level of 10^−3^) might benefit from a change in therapy and being treated more aggressively in an attempt to reach a target of 10^−5^, thereby realizing the benefit from the deeper response. Whilst there is consensus regarding the prognostic application of MRD in MM, there are currently no established guidelines on therapeutic tailoring based on the MRD result. However, it is likely that in future, MRD will indeed guide such therapy decisions as is the case in other haematological malignancies [120–122]. There a many so called response-adaptive clinical trials underway at this time, for example, the PERSEUS phase III randomized study of daratumumab/bortezomib/lenalidomide/dexamethasone versus bortezomib/lenalidomide/dexamethasone in 690 newly diagnosed MM patients (NCT03710603). Patients in the daratumumab arm with sustained MRD negativity (10^−5^) for 12 months after a minimum of 24 months of daratumumab/lenalidomide maintenance therapy will stop daratumumab until progressive disease occurs. Upon recurrence of MRD or loss of CR, patients will re-commence daratumumab.

Until recently, the majority of clinical trials have not included MRD as a primary clinical endpoint. This is largely due to a number of MRD aspects that remain debated, including (1) patient selection (those in sCR and CR or also VGPR), (2) the timing of MRD testing during the treatment course, (3) the optimal cut-off (10^−5^ or 10^−6^), (4) the frequency of MRD evaluation after a MRD negative result, (5) the likelihood and interpretation of false positive and false negative results, (6) testing MRD in bone marrow and also blood, (7) combining different MRD techniques such as NGS and imaging, (8) evaluation of the quantitative tumour burden and further stratification for MRD positive patients, and (9) the evaluation of additional prognostic biomarkers that may complement the MRD result. The standard PFS and OS endpoints provide the most unambiguous evidence for the efficacy of a new therapy for MM; however, current improvements in MM management have considerably prolonged both PFS and OS, making prospective clinical trials lengthy and costly. Therefore, early biomarkers of efficacy that can reliably recapitulate PFS and OS are needed. MRD could be the answer here and there are many trials either planned or currently underway that set MRD as an exclusive or additional primary endpoint to PFS and OS (Table 5). Recently, a large meta-analysis that included data from six randomised trials (3283 newly diagnosed MM patients) confirmed that achievement of MRD negativity strongly correlated with prolonged PFS [123]. Thus, MRD met the Prentice criteria for PFS surrogacy [124].

**Table 5:** Selected phase III trials that include MRD negativity rate as a primary clinical endpoint

Identifier	Regimen	Subjects	Sensitivity/method	Status
NCT03948035	Elo-KRd versus KRd prior to and after ASCT and maintenance with Elo-R versus R	576 NDMM eligible for ASCT	NR/MFC	Recruiting
NCT03652064	Dara-VRd followed by Dara-Rd versus VRd followed by Rd	395 NDMM patients for whom ASCT is not planned as initial therapy	10^–5^/NGS	Active, not recruiting
NCT03617731	Isa-RVd versus RVd for induction, ASCT and Isa-R versus R for maintenance therapy	662 NDMM eligible for ASCT	10^–5^/NGF	Active, not recruiting
NCT04751877	Isa-RVd versus Isa-Rd	270 NDMM non-eligible for ASCT	10^–5^/NGS	Recruiting

Dara, Daratumumab; d, Dexamethasone; Elo, Elotuzumab; Isa, Isatuximab; K, Carfilzomib; R, Lenalidomide; V, Bortezomib; ASCT Autologous stem cell transplantation; NGF, next generation flow cytometry; NGS, next generation sequencing; MFC, multiparameter flow cytometry; NR, not reported; NDMM, newly diagnosed MM

To date, the majority of MRD data have come from retrospective or subset analyses of patients enrolled in clinical trials with few studies examining the prognostic impact of MRD in patients treated in a real-world clinical setting [125–127]. A single institution’s experience with MRD evaluation by NGS in newly diagnosed MM (NDMM) and relapsed/refractory MM (RRMM) patients receiving frontline or salvage therapy was inspiring [128]. The findings suggest that MRD dynamics may play an important role in future therapeutic decision-making. Those achieving MRD negativity at 10^−6^, as well as 10^−5^, had superior median PFS. In the NDMM cohort, 40% of the patients achieved MRD negativity at 10^−6^ and 59% at 10^−5^. Median PFS in the NDMM cohort was superior in those achieving MRD at 10^−5^ versus < 10^−5^ (PFS: 87 months vs 32 months; *P* < 0.001). In the RRMM cohort, 36% achieved MRD negativity at 10^−6^ and 47% at 10^−5^. Median PFS was superior for the RRMM achieving MRD at 10^−5^ versus < 10^−5^ (PFS: 42 months vs 17 months; *P* < 0.01). Serial MRD monitoring identified three categories of NDMM patients: (A) patients with ≥ 3 MRD 10^−6^ negative samples, (B) patients with detectable but continuously declining clonal numbers and (C) patients with stable or increasing clonal number (≥ 1 log). PFS was superior in groups A and B versus C (median PFS not reached, not reached, 55 months, respectively; *P* < 0.001). This retrospective evaluation of MRD used as part of clinical care validates MRD as an important prognostic biomarker in NDMM and RRMM and supports its use as an endpoint in future clinical trials, as well as for clinical decision-making.

## Predictive biomarker-driven precision medicine approaches

Precision or personalised medicine entails managing an individual patient according to their own tumour biology and/or risk profile. The aforementioned section on MRD provided examples of MRD directed therapeutic approaches; however, this can be taken further with therapies selected based on the presence of specific tumour mutations, GEPs, proteins, etc. Some of these “actionable” mutations, proteins, etc., have been discussed previously in the relevant sections of this review; however, those that are translationally promising or in clinical trials are discussed here.

Single agent and combination trials with venetoclax (ABT-199), a small molecule inhibitor of Bcl-2, have consistently demonstrated superior PFS in patients with RRMM harbouring t(11;14) and related increased expression of Bcl-2 gene and protein [57, 129]. Single-agent venetoclax in multiply relapsed/refractory MM patients demonstrated an ORR of 40% among those with t(11;14), whilst *BCL2*:*MCL1* and *BCL2*:*BCL2L1* mRNA expression levels correlated with responses and with t(11;14) status [129]. Another study examined venetoclax in combination with bortezomib and dexamethasone demonstrating an ORR of 67% in all patients and 78% in t(11;14) patients; those with higher *BCL2* expression had deeper responses and longer PFS [130]. The high efficacy of the combination in patients without the t(11;14) or high Bcl-2 expression was speculated to be due to bortezomib upregulating Noxa, a proapoptotic factor that neutralizes Mcl-1, resulting in an increased *BCL2*:*MCL1* ratio and sensitivity to venetoclax [131]. Most recently, the phase III Bellini trial comparing venetoclax, bortezomib and dexamethasone to bortezomib and dexamethasone alone in relapsed/refractory MM patients demonstrated a median PFS of 22 months versus 12 months [57]. Moreover, the ORR was 82% versus 68% (all patients), 90% versus 47% (t(11;14) positive patients) and 85% versus 75% (high *BCL2* expression). However, an excess of infection-related deaths occurred in the venetoclax arm albeit in patients without t(11;14). Whilst initially these findings suggest that moving forward with venetoclax may not be limited to the t(11;14) subgroup when used in combination with a proteasome inhibitor and an assay measuring *BCL2*:*MCL1* or *BCL2*:*BCL2L1* mRNA expression ratios, the Bellini trial has cast doubt on this, with future venetoclax studies focusing on t(11;14) positive MM.

Since the MAPK pathway can be dysregulated due to activating mutations in *NRAS*, *KRAS* or *BRAF* in 40–50% of MM patients (25% *NRAS*, 25% *KRAS* and 4% *BRAF*), BRAF inhibitors such as vemurafenib and dabrafenib, and MEK inhibitors such as trametinib and cobimetinib are being evaluated. In a BRAF V600E-positive RRMM patient with extramedullary disease, vemurafenib administration resulted in a durable response [132]. Similarly, over half of the patients with RAS-mutated MM treated with the MEK inhibitor trametinib experienced at least a 25% reduction in paraprotein although many responses were short lived, especially when the drugs were used as single agents [133]. Whilst most studies to date are case reports and case series, several prospective studies are underway (e.g. MEK inhibition in NCT02834364 and BRAF/NRAS/KRAS inhibition in NCT02407509 and NCT01989598).

Other targets have been identified. *FGFR3*, together with *MMSET/WHSC1*, is overexpressed in 10–15% of MM patients due to the t(4;14) translocation; however, first generation FGFR inhibitors have demonstrated limited efficacy [134]. The recent discovery of activating FGFR3 mutations in a third of t(4;14) cases has renewed interest in novel FGFR inhibitors targeting FGFR3-mutated MM [135, 136]. Similarly, the identification of activating alterations in IDH1/2 and NTRK1/2/3 makes them interesting as potential candidates for precision medicine trials in MM [137–140], as does loss of heterozygosity or ATM/ATR mutations and PARP inhibitors [141]. AMG176 (NCT02675452) and AZD5991 (NCT03218683) inhibitors against anti-apoptotic molecule Mcl-1, the most frequently expressed Bcl-2 family member, are also undergoing clinical evaluation. Given Mcl-1 resides on chromosome 1q, which is frequently amplified in MM, it will be interesting to see whether the new Mcl-1 inhibitors achieve responses in this and other molecular sub-groups of MM patients. Furthermore, translocations resulting in MYC overexpression have been detected in approximately 15% of MM cases and novel approaches aimed at targeting this transcription factor are being tested clinically [142].

Traditional clinical trials are not optimal for the rapid evaluation of precision medicine approaches. Recently, integrated platform trials known as master protocols have been developed to facilitate the simultaneous and rapid testing of multiple treatments in the same trial [143]. An example is the MMRF Myeloma-Developing Regimens Using Genomics (MyDRUG) study (NCT03732703). This is a Phase I/II trial master protocol designed to develop novel precision medicine combinations for patients with high-risk MM whose disease has rapidly progressed in spite of standard-of-care therapies. Genomic alterations that can be therapeutically targeted are being identified with study arms for individuals with RAS/RAF mutations, CDK activating alterations, FGFR3 and IDH2 mutations, as well as t(11;14) translocations. Patients without these genetic aberrations receive an immunotherapy (daratumumab) and all patients receive a backbone of ixazomib, pomalidomide and dexamethasone (IPd). However, a major problem for precision medicine approaches is that of tumour clonal heterogeneity [71]. Specifically, therapy targeting molecular aberrations present in only a subset of MM cells might not achieve clinical benefit and may result in the rapid growth of sub-clones. With regard to the MyDRUG trial, the genetic lesion being targeted must be present in a relatively large proportion of the MM cells, having an allelic fraction > 0.3.

Despite these advances, a number of obstacles remain. Precision medicine MM drugs will require appropriate companion diagnostic tests to detect the targetable lesions, however, assay availability and cost remain barriers. The FDA recently approved the MSK-IMPACT™ and FoundationOne CDx™ panels which will hopefully help to accelerate reimbursement for clinical sequencing. Moreover, assays need to be developed to detect functional changes such as signalling pathway activation rather than only the presence or absence of a genetic or protein lesion. Additionally, bone marrow biopsies in MM are limited in their ability to fully represent the genetic heterogeneity of the disease. As discussed previously, liquid biopsies are emerging as promising tools in MM to satisfy the requirements of precision medicine approaches; however, much work is needed to optimise this methodology for use clinically [100]. Finally, in recent years, new immunological treatments including anti-CD38 and anti-SLAMF7 monoclonal antibodies have become available [4, 144] and B-cell maturation antigen is currently being investigated clinically through a variety of chimeric antigen receptor T-cell (CAR-T) and monoclonal antibody therapeutics [84]. However, reliable methods to evaluate a patient’s immune system are not available, which is frequently deranged in MM patients, particularly those heavily pre-treated. Immune profiling will therefore be essential to match patients with the appropriate combinations of targeted and/or immune-based therapies.

## Conclusion

There continues to be much interest and indeed much progress in elucidating biomarkers that assist with determining prognosis and treatment selection for patients with MM, with the future moving towards precision medicine and individualised patient management. It may be that an integrated approach which includes clinical, serological, imaging, genetic and protein biomarkers is required to guide both therapy selection, as well as prognostication, and ongoing efforts are incorporating new biomarkers such as miRNAs, non-coding RNA and splicing events. Moreover, the presence of functional events such as downstream activating mutations in key signalling pathways, as seen with *KRAS* and *BRAF* mutations in the MAPK pathway, is being considered. Importantly, it will also be necessary to understand the dynamic and changing clinical impact of multiple clones, sub-clones, their evolution and the molecular mechanisms driving these clones, on disease outcome in each MM patient.

The ever-increasing number and complexity of MM drug classes, including immunotherapeutic approaches such as bi-specific monoclonal antibodies, antibody–drug conjugates and CAR-T cells that not only target the malignant plasma cell but also harness the immune system have markedly improved response rates in MM patients. Ultimately, clinically useful biomarkers will need to be relevant in the era of such novel therapies, which in some instances can overcome the poor prognosis of MM patients harbouring traditionally poor prognostic biology. Moreover, to improve upon the tremendous progress made so far, high-throughput technologies are being incorporated into research with array-based methods giving way to sequencing-based methods. In parallel, new bioinformatics methodologies are being developed for detailed analyses of the large amount of data generated. Therefore, the field of MM biomarker development is rapidly progressing, spurred on by new technologies and therapeutic approaches underpinned by a deeper understanding of MM biology with individualised patient management the goal.

## Data Availability

There is nothing to declare.

## References

[1] Rollig C, Knop S, Bornhauser M (2015). Multiple myeloma. Lancet.

[2] Kumar SK, Dispenzieri A, Lacy MQ (2014). Continued improvement in survival in multiple myeloma: changes in early mortality and outcomes in older patients. Leukemia.

[3] Morgan GJ, Walker BA, Davies FE (2012). The genetic architecture of multiple myeloma. Nat Rev Cancer.

[4] Wallington-Beddoe CT, Sobieraj-Teague M, Kuss BJ, Pitson SM (2018). Resistance to proteasome inhibitors and other targeted therapies in myeloma. Br J Haematol.

[5] Biomarkers Definitions Working G. Biomarkers and surrogate endpoints: preferred definitions and conceptual framework. Clin Pharmacol Ther. 2001;69(3):89–95. 10.1067/mcp.2001.113989 11240971

[6] Strimbu K, Tavel JA (2010). What are biomarkers?. Curr Opin HIV AIDS.

[7] Rajkumar SV, Dimopoulos MA, Palumbo A (2014). International myeloma working group updated criteria for the diagnosis of multiple myeloma. Lancet Oncol.

[8] Durie BG, Salmon SE (1975). A clinical staging system for multiple myeloma. Correlation of measured myeloma cell mass with presenting clinical features, response to treatment, and survival. Cancer.

[9] Greipp PR, San Miguel J, Durie BG (2005). International staging system for multiple myeloma. J Clin Oncol.

[10] Palumbo A, Avet-Loiseau H, Oliva S (2015). Revised international staging system for multiple myeloma: a report from international myeloma working group. J Clin Oncol.

[11] Kumar S, Paiva B, Anderson KC (2016). International myeloma working group consensus criteria for response and minimal residual disease assessment in multiple myeloma. Lancet Oncol.

[12] Kyle RA, Remstein ED, Therneau TM (2007). Clinical course and prognosis of smoldering (asymptomatic) multiple myeloma. N Engl J Med.

[13] Perez-Persona E, Vidriales MB, Mateo G (2007). New criteria to identify risk of progression in monoclonal gammopathy of uncertain significance and smoldering multiple myeloma based on multiparameter flow cytometry analysis of bone marrow plasma cells. Blood.

[14] Mateos MV, Hernandez MT, Giraldo P (2013). Lenalidomide plus dexamethasone for high-risk smoldering multiple myeloma. N Engl J Med.

[15] Lakshman A, Rajkumar SV, Buadi FK (2018). Risk stratification of smoldering multiple myeloma incorporating revised IMWG diagnostic criteria. Blood Cancer J.

[16] Dispenzieri A, Kyle RA, Katzmann JA (2008). Immunoglobulin free light chain ratio is an independent risk factor for progression of smoldering (asymptomatic) multiple myeloma. Blood.

[17] Rajkumar SV, Kyle RA, Therneau TM (2004). Presence of monoclonal free light chains in the serum predicts risk of progression in monoclonal gammopathy of undetermined significance. Br J Haematol.

[18] El Naggar AA, El-Naggar M, el Mokhamer H, Avad MW (2015). Prognostic value of serum free light chain in multiple myeloma. Egypt J Immunol.

[19] Barley K, Tindle S, Bagiella E, Jagannath S, Chari A (2015). Serum free light chain assessment early after stem cell transplantation as a prognostic factor in multiple myeloma. Clin Lymphoma Myeloma Leuk.

[20] Ozkurt ZN, Sucak GT, Aki SZ, Yagci M, Haznedar R (2017). Early prognostic value of monitoring serum free light chain in patients with multiple myeloma undergoing autologous stem cell transplantation. Cancer Invest.

[21] Kumar S, Zhang L, Dispenzieri A (2010). Relationship between elevated immunoglobulin free light chain and the presence of IgH translocations in multiple myeloma. Leukemia.

[22] Brioli A, Giles H, Pawlyn C (2014). Serum free immunoglobulin light chain evaluation as a marker of impact from intraclonal heterogeneity on myeloma outcome. Blood.

[23] Tacchetti P, Cavo M, Rocchi S (2016). Prognostic impact of serial measurements of serum-free light chain assay throughout the course of newly diagnosed multiple myeloma treated with bortezomib-based regimens. Leuk Lymphoma.

[24] Tacchetti P, Pezzi A, Zamagni E (2017). Role of serum free light chain assay in the detection of early relapse and prediction of prognosis after relapse in multiple myeloma patients treated upfront with novel agents. Haematologica.

[25] Hillengass J, Moulopoulos LA, Delorme S (2017). Whole-body computed tomography versus conventional skeletal survey in patients with multiple myeloma: a study of the international myeloma working group. Blood Cancer J.

[26] Durie BG, Waxman AD, D'Agnolo A, Williams CM (2002). Whole-body (18)F-FDG PET identifies high-risk myeloma. J Nucl Med.

[27] Antoch G, Vogt FM, Freudenberg LS (2003). Whole-body dual-modality PET/CT and whole-body MRI for tumor staging in oncology. JAMA.

[28] Hillengass J, Usmani S, Rajkumar SV (2019). International myeloma working group consensus recommendations on imaging in monoclonal plasma cell disorders. Lancet Oncol.

[29] Dimopoulos MA, Hillengass J, Usmani S (2015). Role of magnetic resonance imaging in the management of patients with multiple myeloma: a consensus statement. J Clin Oncol.

[30] Hillengass J, Fechtner K, Weber MA (2010). Prognostic significance of focal lesions in whole-body magnetic resonance imaging in patients with asymptomatic multiple myeloma. J Clin Oncol.

[31] Dammacco F, Rubini G, Ferrari C, Vacca A, Racanelli V (2015). (1)(8)F-FDG PET/CT: a review of diagnostic and prognostic features in multiple myeloma and related disorders. Clin Exp Med.

[32] Fonti R, Larobina M, Del Vecchio S (2012). Metabolic tumor volume assessed by 18F-FDG PET/CT for the prediction of outcome in patients with multiple myeloma. J Nucl Med.

[33] Mesguich C, Fardanesh R, Tanenbaum L, Chari A, Jagannath S, Kostakoglu L (2014). State of the art imaging of multiple myeloma: comparative review of FDG PET/CT imaging in various clinical settings. Eur J Radiol.

[34] Zamagni E, Nanni C, Mancuso K (2015). PET/CT improves the definition of complete response and allows to detect otherwise unidentifiable skeletal progression in multiple myeloma. Clin Cancer Res.

[35] Zamagni E, Patriarca F, Nanni C (2011). Prognostic relevance of 18-F FDG PET/CT in newly diagnosed multiple myeloma patients treated with up-front autologous transplantation. Blood.

[36] Patel CG, Yee AJ, Scullen TA (2014). Biomarkers of bone remodeling in multiple myeloma patients to tailor bisphosphonate therapy. Clin Cancer Res.

[37] Terpos E, Dimopoulos MA, Sezer O (2010). The use of biochemical markers of bone remodeling in multiple myeloma: a report of the international myeloma working group. Leukemia.

[38] Abildgaard N, Brixen K, Kristensen JE, Eriksen EF, Nielsen JL, Heickendorff L (2003). Comparison of five biochemical markers of bone resorption in multiple myeloma: elevated pre-treatment levels of S-ICTP and U-Ntx are predictive for early progression of the bone disease during standard chemotherapy. Br J Haematol.

[39] Corso A, Arcaini L, Mangiacavalli S (2001). Biochemical markers of bone disease in asymptomatic early stage multiple myeloma. A study on their role in identifying high risk patients. Haematologica.

[40] Jakob C, Zavrski I, Heider U (2002). Bone resorption parameters [carboxy-terminal telopeptide of type-I collagen (ICTP), amino-terminal collagen type-I telopeptide (NTx), and deoxypyridinoline (Dpd)] in MGUS and multiple myeloma. Eur J Haematol.

[41] Jakob C, Sterz J, Liebisch P (2008). Incorporation of the bone marker carboxy-terminal telopeptide of type-1 collagen improves prognostic information of the international staging system in newly diagnosed symptomatic multiple myeloma. Leukemia.

[42] Schutt P, Rebmann V, Brandhorst D (2008). The clinical significance of soluble human leukocyte antigen class-I, ICTP, and RANKL molecules in multiple myeloma patients. Hum Immunol.

[43] Terpos E, Politou M, Szydlo R (2004). Autologous stem cell transplantation normalizes abnormal bone remodeling and sRANKL/osteoprotegerin ratio in patients with multiple myeloma. Leukemia.

[44] Christenson RH (1997). Biochemical markers of bone metabolism: an overview. Clin Biochem.

[45] Munshi NC, Anderson KC, Bergsagel PL (2011). Consensus recommendations for risk stratification in multiple myeloma: report of the international myeloma workshop consensus panel 2. Blood.

[46] Fonseca R, Barlogie B, Bataille R (2004). Genetics and cytogenetics of multiple myeloma: a workshop report. Cancer Res.

[47] Gonzalez D, van der Burg M, Garcia-Sanz R (2007). Immunoglobulin gene rearrangements and the pathogenesis of multiple myeloma. Blood.

[48] Chretien ML, Corre J, Lauwers-Cances V (2015). Understanding the role of hyperdiploidy in myeloma prognosis: which trisomies really matter?. Blood.

[49] Vu T, Gonsalves W, Kumar S (2015). Characteristics of exceptional responders to lenalidomide-based therapy in multiple myeloma. Blood Cancer J.

[50] Fonseca R, Bergsagel PL, Drach J (2009). International Myeloma Working Group molecular classification of multiple myeloma: spotlight review. Leukemia.

[51] Zojer N, Konigsberg R, Ackermann J (2000). Deletion of 13q14 remains an independent adverse prognostic variable in multiple myeloma despite its frequent detection by interphase fluorescence in situ hybridization. Blood.

[52] Avet-Loiseau H, Leleu X, Roussel M (2010). Bortezomib plus dexamethasone induction improves outcome of patients with t(4;14) myeloma but not outcome of patients with del(17p). J Clin Oncol.

[53] San Miguel JF, Schlag R, Khuageva NK (2008). Bortezomib plus melphalan and prednisone for initial treatment of multiple myeloma. N Engl J Med.

[54] Boyd KD, Ross FM, Walker BA (2011). Mapping of chromosome 1p deletions in myeloma identifies FAM46C at 1p12 and CDKN2C at 1p32.3 as being genes in regions associated with adverse survival. Clin Cancer Res.

[55] Fonseca R, Blood EA, Oken MM (2002). Myeloma and the t(11;14)(q13;q32); evidence for a biologically defined unique subset of patients. Blood.

[56] Avet-Loiseau H, Attal M, Moreau P (2007). Genetic abnormalities and survival in multiple myeloma: the experience of the Intergroupe Francophone du Myelome. Blood.

[57] Kumar SK, Harrison SJ, Cavo M (2020). Venetoclax or placebo in combination with bortezomib and dexamethasone in patients with relapsed or refractory multiple myeloma (BELLINI): a randomised, double-blind, multicentre, phase 3 trial. Lancet Oncol.

[58] Sonneveld P, Schmidt-Wolf IG, van der Holt B (2012). Bortezomib induction and maintenance treatment in patients with newly diagnosed multiple myeloma: results of the randomized phase III HOVON-65/ GMMG-HD4 trial. J Clin Oncol.

[59] Qiang YW, Ye S, Chen Y (2016). MAF protein mediates innate resistance to proteasome inhibition therapy in multiple myeloma. Blood.

[60] Richardson PG, Weller E, Lonial S (2010). Lenalidomide, bortezomib, and dexamethasone combination therapy in patients with newly diagnosed multiple myeloma. Blood.

[61] Kumar S, Fonseca R, Ketterling RP (2012). Trisomies in multiple myeloma: impact on survival in patients with high-risk cytogenetics. Blood.

[62] Szalat R, Avet-Loiseau H, Munshi NC (2016). Gene expression profiles in myeloma: ready for the real world?. Clin Cancer Res.

[63] Shaughnessy JD, Zhan F, Burington BE (2007). A validated gene expression model of high-risk multiple myeloma is defined by deregulated expression of genes mapping to chromosome 1. Blood.

[64] Decaux O, Lode L, Magrangeas F (2008). Prediction of survival in multiple myeloma based on gene expression profiles reveals cell cycle and chromosomal instability signatures in high-risk patients and hyperdiploid signatures in low-risk patients: a study of the Intergroupe Francophone du Myelome. J Clin Oncol.

[65] Zandecki M, Lai JL, Facon T (1996). Multiple myeloma: almost all patients are cytogenetically abnormal. Br J Haematol.

[66] Hose D, Reme T, Hielscher T (2011). Proliferation is a central independent prognostic factor and target for personalized and risk-adapted treatment in multiple myeloma. Haematologica.

[67] Kuiper R, Broyl A, de Knegt Y (2012). A gene expression signature for high-risk multiple myeloma. Leukemia.

[68] Dickens NJ, Walker BA, Leone PE (2010). Homozygous deletion mapping in myeloma samples identifies genes and an expression signature relevant to pathogenesis and outcome. Clin Cancer Res.

[69] van Beers EH, van Vliet MH, Kuiper R (2017). Prognostic validation of SKY92 and its combination with ISS in an independent cohort of patients with multiple myeloma. Clin Lymphoma Myeloma Leuk.

[70] Chapman MA, Lawrence MS, Keats JJ (2011). Initial genome sequencing and analysis of multiple myeloma. Nature.

[71] Lohr JG, Stojanov P, Carter SL (2014). Widespread genetic heterogeneity in multiple myeloma: implications for targeted therapy. Cancer Cell.

[72] Walker BA, Boyle EM, Wardell CP (2015). Mutational spectrum, copy number changes, and outcome: results of a sequencing study of patients with newly diagnosed myeloma. J Clin Oncol.

[73] Bjorklund CC, Lu L, Kang J (2015). Rate of CRL4(CRBN) substrate Ikaros and Aiolos degradation underlies differential activity of lenalidomide and pomalidomide in multiple myeloma cells by regulation of c-Myc and IRF4. Blood Cancer J.

[74] Chen L, Wang S, Zhou Y (2010). Identification of early growth response protein 1 (EGR-1) as a novel target for JUN-induced apoptosis in multiple myeloma. Blood.

[75] Zhu YX, Braggio E, Shi CX (2014). Identification of cereblon-binding proteins and relationship with response and survival after IMiDs in multiple myeloma. Blood.

[76] Walker BA, Mavrommatis K, Wardell CP (2019). A high-risk, double-hit, group of newly diagnosed myeloma identified by genomic analysis. Leukemia.

[77] Dytfeld D, Luczak M, Wrobel T (2016). Comparative proteomic profiling of refractory/relapsed multiple myeloma reveals biomarkers involved in resistance to bortezomib-based therapy. Oncotarget.

[78] Dytfeld D, Rosebeck S, Kandarpa M (2015). Proteomic profiling of naive multiple myeloma patient plasma cells identifies pathways associated with favourable response to bortezomib-based treatment regimens. Br J Haematol.

[79] Zatula A, Dikic A, Mulder C (2017). Proteome alterations associated with transformation of multiple myeloma to secondary plasma cell leukemia. Oncotarget.

[80] Glavey SV, Naba A, Manier S (2017). Proteomic characterization of human multiple myeloma bone marrow extracellular matrix. Leukemia.

[81] Huang SY, Lin HH, Lin CW (2016). Soluble PD-L1: a biomarker to predict progression of autologous transplantation in patients with multiple myeloma. Oncotarget.

[82] Usmani SZ, Schjesvold F, Oriol A (2019). Pembrolizumab plus lenalidomide and dexamethasone for patients with treatment-naive multiple myeloma (KEYNOTE-185): a randomised, open-label, phase 3 trial. Lancet Haematol.

[83] Mateos MV, Blacklock H, Schjesvold F (2019). Pembrolizumab plus pomalidomide and dexamethasone for patients with relapsed or refractory multiple myeloma (KEYNOTE-183): a randomised, open-label, phase 3 trial. Lancet Haematol.

[84] Yu B, Jiang T, Liu D (2020). BCMA-targeted immunotherapy for multiple myeloma. J Hematol Oncol.

[85] Ghermezi M, Li M, Vardanyan S (2017). Serum B-cell maturation antigen: a novel biomarker to predict outcomes for multiple myeloma patients. Haematologica.

[86] Xie Z, Gunaratne J, Cheong LL (2013). Plasma membrane proteomics identifies biomarkers associated with MMSET overexpression in T(4;14) multiple myeloma. Oncotarget.

[87] Ishibashi M, Soeda S, Sasaki M (2018). Clinical impact of serum soluble SLAMF7 in multiple myeloma. Oncotarget.

[88] Lonial S, Dimopoulos M, Palumbo A (2015). Elotuzumab therapy for relapsed or refractory multiple myeloma. N Engl J Med.

[89] Gandhi AK, Mendy D, Waldman M (2014). Measuring cereblon as a biomarker of response or resistance to lenalidomide and pomalidomide requires use of standardized reagents and understanding of gene complexity. Br J Haematol.

[90] Thakurta A, Gandhi AK, Waldman MF (2014). Absence of mutations in cereblon (CRBN) and DNA damage-binding protein 1 (DDB1) genes and significance for IMiD therapy. Leukemia.

[91] Barwick BG, Neri P, Bahlis NJ (2019). Multiple myeloma immunoglobulin lambda translocations portend poor prognosis. Nat Commun.

[92] Bennett MK, Wallington-Beddoe CT, Pitson SM (2019). Sphingolipids and the unfolded protein response. Biochim Biophys Acta Mol Cell Biol Lipids.

[93] Mynott RL, Wallington-Beddoe CT (2021). Drug and solute transporters in mediating resistance to novel therapeutics in multiple myeloma. ACS Pharmacol Transl Sci.

[94] Mynott RL, Wallington-Beddoe CT (2021). Inhibition of P-glycoprotein does not increase the efficacy of proteasome inhibitors in multiple myeloma cells. ACS Pharmacol Transl Sci.

[95] Ebert LM, Vandyke K, Johan MZ (2021). Desmoglein-2 expression is an independent predictor of poor prognosis patients with multiple myeloma. Mol Oncol.

[96] Chakraborty R, Muchtar E, Kumar SK (2016). Risk stratification in myeloma by detection of circulating plasma cells prior to autologous stem cell transplantation in the novel agent era. Blood Cancer J.

[97] Gonsalves WI, Morice WG, Rajkumar V (2014). Quantification of clonal circulating plasma cells in relapsed multiple myeloma. Br J Haematol.

[98] Gonsalves WI, Rajkumar SV, Gupta V (2014). Quantification of clonal circulating plasma cells in newly diagnosed multiple myeloma: implications for redefining high-risk myeloma. Leukemia.

[99] Lohr JG, Kim S, Gould J (2016). Genetic interrogation of circulating multiple myeloma cells at single-cell resolution. Sci Transl Med..

[100] Manier S, Park J, Capelletti M (2018). Whole-exome sequencing of cell-free DNA and circulating tumor cells in multiple myeloma. Nat Commun.

[101] Mishima Y, Paiva B, Shi J (2017). The mutational landscape of circulating tumor cells in multiple myeloma. Cell Rep.

[102] Jelinek T, Bezdekova R, Zatopkova M (2017). Current applications of multiparameter flow cytometry in plasma cell disorders. Blood Cancer J.

[103] Sanoja-Flores L, Flores-Montero J, Garces JJ (2018). Next generation flow for minimally-invasive blood characterization of MGUS and multiple myeloma at diagnosis based on circulating tumor plasma cells (CTPC). Blood Cancer J.

[104] Huhn S, Weinhold N, Nickel J (2017). Circulating tumor cells as a biomarker for response to therapy in multiple myeloma patients treated within the GMMG-MM5 trial. Bone Marrow Transplant.

[105] Kis O, Kaedbey R, Chow S (2017). Circulating tumour DNA sequence analysis as an alternative to multiple myeloma bone marrow aspirates. Nat Commun.

[106] Biancon G, Gimondi S, Vendramin A, Carniti C, Corradini P (2018). Noninvasive molecular monitoring in multiple myeloma patients using cell-free tumor DNA: a pilot study. J Mol Diagn.

[107] Mazzotti C, Buisson L, Maheo S (2018). Myeloma MRD by deep sequencing from circulating tumor DNA does not correlate with results obtained in the bone marrow. Blood Adv.

[108] Oberle A, Brandt A, Voigtlaender M (2017). Monitoring multiple myeloma by next-generation sequencing of V(D)J rearrangements from circulating myeloma cells and cell-free myeloma DNA. Haematologica.

[109] Guo G, Raje NS, Seifer C (2018). Genomic discovery and clonal tracking in multiple myeloma by cell-free DNA sequencing. Leukemia.

[110] Mithraprabhu S, Sirdesai S, Chen M, Khong T, Spencer A (2018). Circulating tumour DNA analysis for tumour genome characterisation and monitoring disease burden in extramedullary multiple myeloma. Int J Mol Sci.

[111] Paiva B, Puig N, Cedena MT (2020). Measurable residual disease by next-generation flow cytometry in multiple myeloma. J Clin Oncol.

[112] Perrot A, Lauwers-Cances V, Corre J (2018). Minimal residual disease negativity using deep sequencing is a major prognostic factor in multiple myeloma. Blood.

[113] Kostopoulos IV, Ntanasis-Stathopoulos I, Gavriatopoulou M, Tsitsilonis OE, Terpos E (2020). Minimal residual disease in multiple myeloma: current landscape and future applications with immunotherapeutic approaches. Front Oncol.

[114] Flores-Montero J, Sanoja-Flores L, Paiva B (2017). Next Generation Flow for highly sensitive and standardized detection of minimal residual disease in multiple myeloma. Leukemia.

[115] Lahuerta JJ, Paiva B, Vidriales MB (2017). Depth of response in multiple myeloma: a pooled analysis of three PETHEMA/GEM clinical trials. J Clin Oncol.

[116] Munshi NC, Avet-Loiseau H, Rawstron AC (2017). Association of minimal residual disease with superior survival outcomes in patients with multiple myeloma: a meta-analysis. JAMA Oncol.

[117] Ladetto M, Pagliano G, Ferrero S (2010). Major tumor shrinking and persistent molecular remissions after consolidation with bortezomib, thalidomide, and dexamethasone in patients with autografted myeloma. J Clin Oncol.

[118] Jackson GH, Davies FE, Pawlyn C (2019). Lenalidomide maintenance versus observation for patients with newly diagnosed multiple myeloma (Myeloma XI): a multicentre, open-label, randomised, phase 3 trial. Lancet Oncol.

[119] Fulciniti M, Munshi NC, Martinez-Lopez J (2015). Deep response in multiple myeloma: a critical review. Biomed Res Int.

[120] Bassan R, Bruggemann M, Radcliffe HS, Hartfield E, Kreuzbauer G, Wetten S (2019). A systematic literature review and meta-analysis of minimal residual disease as a prognostic indicator in adult B-cell acute lymphoblastic leukemia. Haematologica.

[121] Del Giudice I, Raponi S, Della Starza I (2019). Minimal residual disease in chronic lymphocytic leukemia: a new goal?. Front Oncol.

[122] Izzo B, Gottardi EM, Errichiello S, Daraio F, Barate C, Galimberti S (2019). Monitoring chronic myeloid leukemia: how molecular tools may drive therapeutic approaches. Front Oncol.

[123] Avet-Loiseau H, Ludwig H, Landgren O (2020). Minimal residual disease status as a surrogate endpoint for progression-free survival in newly diagnosed multiple myeloma studies: a meta-analysis. Clin Lymphoma Myeloma Leuk.

[124] Prentice RL (1989). Surrogate endpoints in clinical trials: definition and operational criteria. Stat Med.

[125] Attal M, Lauwers-Cances V, Hulin C (2017). Lenalidomide, bortezomib, and dexamethasone with transplantation for myeloma. N Engl J Med.

[126] Mateos MV, Dimopoulos MA, Cavo M (2018). Daratumumab plus bortezomib, melphalan, and prednisone for untreated myeloma. N Engl J Med.

[127] Palumbo A, Chanan-Khan A, Weisel K (2016). Daratumumab, bortezomib, and dexamethasone for multiple myeloma. N Engl J Med.

[128] Martinez-Lopez J, Wong SW, Shah N (2020). Clinical value of measurable residual disease testing for assessing depth, duration, and direction of response in multiple myeloma. Blood Adv.

[129] Kumar S, Kaufman JL, Gasparetto C (2017). Efficacy of venetoclax as targeted therapy for relapsed/refractory t(11;14) multiple myeloma. Blood.

[130] Moreau P, Chanan-Khan A, Roberts AW (2017). Promising efficacy and acceptable safety of venetoclax plus bortezomib and dexamethasone in relapsed/refractory MM. Blood.

[131] Punnoose EA, Leverson JD, Peale F (2016). Expression profile of BCL-2, BCL-XL, and MCL-1 predicts pharmacological response to the BCL-2 selective antagonist venetoclax in multiple myeloma models. Mol Cancer Ther.

[132] Andrulis M, Lehners N, Capper D (2013). Targeting the BRAF V600E mutation in multiple myeloma. Cancer Discov.

[133] Heuck CJ, Jethava Y, Khan R (2016). Inhibiting MEK in MAPK pathway-activated myeloma. Leukemia.

[134] Scheid C, Reece D, Beksac M (2015). Phase 2 study of dovitinib in patients with relapsed or refractory multiple myeloma with or without t(4;14) translocation. Eur J Haematol.

[135] Chae YK, Ranganath K, Hammerman PS (2017). Inhibition of the fibroblast growth factor receptor (FGFR) pathway: the current landscape and barriers to clinical application. Oncotarget.

[136] Kalff A, Spencer A (2012). The t(4;14) translocation and FGFR3 overexpression in multiple myeloma: prognostic implications and current clinical strategies. Blood Cancer J.

[137] Kortum KM, Mai EK, Hanafiah NH (2016). Targeted sequencing of refractory myeloma reveals a high incidence of mutations in CRBN and Ras pathway genes. Blood.

[138] Joshi SK, Davare MA, Druker BJ, Tognon CE (2019). Revisiting NTRKs as an emerging oncogene in hematological malignancies. Leukemia.

[139] DiNardo CD, Stein EM, de Botton S (2018). Durable remissions with ivosidenib in IDH1-mutated relapsed or refractory AML. N Engl J Med.

[140] Pawlyn C, Kaiser MF, Heuck C (2016). The spectrum and clinical impact of epigenetic modifier mutations in myeloma. Clin Cancer Res.

[141] Pawlyn C, Loehr A, Ashby C (2018). Loss of heterozygosity as a marker of homologous repair deficiency in multiple myeloma: a role for PARP inhibition?. Leukemia.

[142] Jovanovic KK, Roche-Lestienne C, Ghobrial IM, Facon T, Quesnel B, Manier S (2018). Targeting MYC in multiple myeloma. Leukemia.

[143] Woodcock J, LaVange LM (2017). Master protocols to study multiple therapies, multiple diseases, or both. N Engl J Med.

[144] Anderson KC (2018). Promise of immune therapies in multiple myeloma. J Oncol Pract.

